# Impact of a music exercise on oral function in patients with maxillofacial defects: a pilot randomized controlled trial

**DOI:** 10.3389/froh.2026.1786928

**Published:** 2026-10-28

**Authors:** Ziyi Bai, Yuka Sumita, Mariko Hattori, Xuewei Han, Menghan Miao, Xinyu Pang, Gen Tanabe, Noriyuki Wakabayashi

**Affiliations:** 1 Independent Researcher, Tokyo, Japan; 2 Department of Partial and Complete Denture, School of Life Dentistry at Tokyo, The Nippon Dental University, Tokyo, Japan; 3 Department of Advanced Prosthodontics, Graduate School, Medical and Dental Sciences, Institute of Science Tokyo, Tokyo, Japan; 4 Shanghai Ninth People’s Hospital, Shanghai, China; 5 Department of Sports Dentistry, Meikai University School of Dentistry, Sakado, Saitama, Japan

**Keywords:** maxillofacial defect, maxillofacial prosthetics, music exercise, oral exercise, oral function

## Abstract

**Statement of problem:**

Patients with maxillofacial defects, including head and neck cancer survivors, frequently experience impaired oral function that negatively affects quality of life. Although music-based exercise has been reported to improve cognitive and psychological outcomes in older adults, its effects on oral function remain unclear.

**Purpose:**

To investigate the effects of a combined music-and-exercise program on oral function in patients with maxillofacial defects.

**Material and methods:**

A pilot randomized controlled trial was conducted from December 2022 to June 2023. Twenty patients with maxillofacial defects were randomly assigned to either a music-exercise group or a usual-care control group. The intervention consisted of a tablet-based exercise program integrating rhythmic vocalization, consonant articulation exercises, singing, and upper- and lower-limb movements synchronized with music. Oral function was assessed at baseline, 1 month, and 3 months using maximum occlusal force, lip pressure, tongue pressure, oral diadochokinesis, the Repetitive Saliva Swallowing Test, the Simplified Nutritional Appetite Questionnaire, and the cough test.

**Results:**

Seventeen participants completed the study (exercise group, *n* = 8; control group, *n* = 9). In the exercise group, lip pressure increased significantly from 7.72 ± 2.06 N at baseline to 8.95 ± 2.03 N at 1 month (*P* = .02) and 9.17 ± 1.91 N at 3 months (*P* = .004). Maximum occlusal force increased from 268.52 ± 107.13 N to 417.42 ± 170.64 N at 3 months (*P* = .01), and oral diadochokinesis for/pa/ improved from 5.44 ± 0.76 to 5.93 ± 0.75 syllables/s (*P* = .002). No significant within-group improvements were observed in the control group. Greater training time was positively correlated with improvements in maximum occlusal force, lip pressure, and oral diadochokinesis.

**Conclusion:**

This exploratory randomized trial suggests that a combined music-and-exercise program may improve maximum occlusal force, lip pressure, and oral diadochokinesis in patients with maxillofacial defects. However, because of the small sample size, per-protocol analysis, and the absence of a non-music exercise comparison group, these findings should be considered preliminary and require confirmation in larger, adequately powered pilot randomized controlled trials.

**Clinical Trial Registration:**

https://center6.umin.ac.jp/cgi-open-bin/ctr_e/ctr_view.cgi?recptno=R000053438, UMIN-CTR UMIN000046840.

## 1 Introduction

Maxillofacial defects commonly arise following ablative surgery for head and neck cancer (HNC) or from congenital conditions such as cleft lip and palate (CLP). Although these populations differ in etiology and treatment history, both frequently experience impairments in mastication, swallowing, speech, and social communication despite prosthetic rehabilitation. Because most patients with HNC are older adults, age-related oral hypofunction may further aggravate these functional impairments. Therefore, rehabilitation for patients with maxillofacial defects requires a multidisciplinary approach aimed at restoring both function and quality of life (1–3). There has been growing interest in exploring innovative therapeutic interventions in recent years.

Exercise has long been recognized as a beneficial intervention for promoting physical and functional recovery in various medical conditions (4–6). It involves repetitive movements and targeted exercises designed to strengthen muscles, increase range of motion, and improve coordination. Several studies have demonstrated the feasibility of oral exercise in improving oral function in maxillofacial defect patients. Ouyoung et al. reported that speech function in HNC survivors was improved by using a voice rehabilitation program (4). Silbergleit et al. found that HNC survivors who exercise may perceive improvements in diet and swallowing skills (5). Cohen et al. concluded that device-assisted mouth-opening exercise is effective and safe for the management of patients with mandibular defects (6). However, some barriers to exercise, including fear of movement and aggravation of pain, time management issues, and nonadherence or discordance, can adversely affect, or even eliminate, the effectiveness of medical interventions (7, 8). Satoh et al. used the Yamaha Machikado Exercise, a music-based physical exercise program, in elderly people and reported that it produced greater improvements in cognitive function and psychological symptoms than exercise without music (9). Feiss et al. found that exercise was maintained for a longer time in individuals who performed exercise with music than in those who performed exercise without music (10). Music may facilitate rehabilitation through several mechanisms. Rhythmic auditory stimulation has been reported to enhance motor timing and coordination, while musical accompaniment may increase motivation and adherence to repetitive exercise programs (9, 10). Singing also requires coordinated respiratory, phonatory, lingual, and labial movements that are directly relevant to oral functional rehabilitation. However, although previous studies have investigated music-based exercise and oral exercise in older adults and patients with head and neck cancer or maxillofacial defects, the effects of a combined music-and-exercise program on objective oral function in patients with maxillofacial defects have not yet been adequately investigated. Therefore, there is a need to investigate whether incorporating music into an oral exercise program can improve adherence and oral function in patients with maxillofacial defects.

Oral function is influenced by various physiological parameters, each reflects different aspects and provides valuable insights into overall health. The maximum occlusal force reflects the muscle strength of the masseter and temporalis and is related to food intake ability, cognitive function, and mortality (11–13); tongue pressure reflects the tongue muscle force, which aids in food bolus formation and digestion (14); lip pressure, which is controlled by the orbicularis oris muscle, plays a crucial role in food intake and facial expressions (15, 16); oral diadochokinesis (OD) reflects the tongue–lip motor function (11); the repetitive saliva swallowing test (RSST) is a dysphagia screening tool and is a strong predictor of chronic obstructive pulmonary disease (17); the Simplified Nutritional Appetite Questionnaire (SNAQ) assesses appetite and screens for anorexia (18); and cough tests are used to monitor silent aspiration (19). Therefore, evaluating multiple oral function parameters may provide a comprehensive assessment of the potential effects of the intervention.

This study aimed to investigate whether participation in a combined music-and-exercise program was associated with improvements in oral function in patients with maxillofacial defects through a pilot randomized controlled trial. The null hypothesis was that participation in the combined music-and-exercise program would not result in differences in oral function after 3 months of intervention compared with baseline or with the control group receiving usual care.

## 2 Material and methods

### 2.1 Trial design

This was an exploratory pilot randomized, controlled, parallel-group, assessor-blinded trial. Patients were randomly assigned to either the control group (no intervention) or the exercise group (intervention) in a 1:1 ratio. The study was conducted from December 2022 to June 2023. The study's purpose and content were introduced and the recruitment process was conducted by two prosthodontists. A statistician generated the randomization sequence, which was implemented by an exercise instructor who also explained the exercise regimen to the patients. Examiners who were blinded to the outcome of group allocation evaluated oral function and recorded the data.

### 2.2 Patient and public involvement (PPI)

Patients and the public were not involved in the design, conduct, reporting, or dissemination plans of this study. Future iterations will incorporate survivor advisors to optimize feasibility and relevance.

### 2.3 Trial registration

This trial was registered with UMIN-CTR (UMIN000046840) prior to participant enrollment.

### 2.4 Patients and ethics

This trial was carried out in the Clinic for Maxillofacial Prosthetics of Tokyo Medical and Dental University in Japan. The participants were recruited over a 6-month period from December 2022 to February 2023. The inclusion criteria were as follows: [1] age >18 years; [2] history of maxillofacial surgery (either for HNC or CLP); [3] required a definitive prosthesis and had fully adapted to it for >3 months. Patients who satisfied any of the following criteria were excluded from the study: [1] unstable systemic disease; [2] problems in understanding the exercise or unable to perform the exercise by themselves; and [3] received exercise-based treatment after surgery. Each of the participants gave informed consent before being randomly allocated to one of the two groups. The study was in accordance with the CONSORT guidelines and was approved by the Ethics Review Board of Tokyo Medical and Dental University (D2021-096). Any video or identifiable data were handled according to institutional policy; access was restricted to authorized study personnel. The inclusion of patients with both HNC and CLP may introduce clinical heterogeneity. However, all participants shared common characteristics of maxillofacial defects requiring prosthetic rehabilitation, which was the primary focus of this study.

### 2.5 Sample size calculation

The required sample size was calculated using (G*Power 3.1.9.7; Heinrich Heine University Düsseldorf, Düsseldorf, Germany). Lip pressure and tongue pressure were selected as the primary outcomes. Based on preliminary data, the standard deviations were estimated to be 2.7 N for lip pressure and 9 kPa for tongue pressure, with expected between-group differences of 3.2 N and 10 kPa, respectively. Assuming a statistical power of 80% and a two-sided significance level of 0.05, the required sample size was estimated to be 24 participants (12 per group). The original sample size calculation did not incorporate an explicit allowance for participant attrition. Consequently, although 24 participants were recruited, only 17 participants completed the study, which reduced the effective sample size and statistical power.

### 2.6 Randomization

Randomization was performed using JMP version 14 software (SAS Institute Inc.) by an independent statistician. A computer-generated random allocation sequence with a 1:1 allocation ratio was used. Randomization was stratified according to age and sex. Because of the exploratory nature of this pilot study and the limited sample size, defect type (head and neck cancer or cleft lip and palate), surgical extent, and treatment history (e.g., radiotherapy or chemotherapy) were not used as stratification factors. Consequently, some baseline imbalance between groups may have occurred despite random allocation.

### 2.7 Intervention

After providing informed consent, the participants were allocated to their group but were unaware of their group allocation. In an independent room, the exercise instructor informed the participants of their group assignment according to the randomization sequence. In the control group, participants received regular dental care. In the exercise group, participants received instructions about the exercise with music program in addition to the usual dental care. Each patient in the exercise group was given a tablet computer (iPad 9; Apple Inc.) with an exercise application based on the Yamaha Machikado Exercise (physical exercise with music) (9). The exercise instructor provided instructions on using the tablet computer and a booklet with written and illustrated instructions. The camera of the tablet computer recorded the participants’ exercises to help correct movements. The software recorded the total training time, which represented how long the participants performed the exercise. The participants were instructed to do the exercises daily, choose from different exercise courses, and sing the selected songs. They brought the tablet computer back to the clinic for follow-up visits and were encouraged to call the clinic if they encountered any problems.

Participants were instructed to complete at least one training session per day. Adherence was monitored using application-generated logs of training frequency and cumulative training time. The exercise protocol was as follows. [1] Maximum phonation time: saying the vowel/a/ for as long as possible in one breath. [2] Courses: the 6-min course provides practice in pronouncing the five vowels/a/,/i/,/u/,/e/,/o/ in different speeds, in different orders, and in different combinations with background music rhythm; the 9-min course contains the same contents as the 6-min course plus consonant pronunciation, consonant articulation training consisted of repeated production of/pa/,/ta/, and/ka/, targeting lip closure, anterior tongue movement, and posterior tongue movement, respectively; the 24-min course contains the contents of the 9-min course plus limb exercises. The limb exercise component included synchronized upper-extremity movements performed in time with the musical rhythm, such as shoulder flexion, elbow extension, and rhythmic hand movements. The participants were asked to choose one of the three courses by themselves. [3] Songs: five pre-recorded songs with standardized rhythmic accompaniment were incorporated into the application. Participants were allowed to select their preferred song from the available library to enhance engagement. Song selection was recorded automatically by the application. [4] Maximum phonation time is measured again. Participants selected among the available exercise durations (6, 9, or 24 min) according to individual preference. This pragmatic approach was adopted to maximize adherence; however, it may have introduced variability in exercise dosage.

### 2.8 Blinding

Due to the nature of the intervention, participants were not blinded to group allocation. However, outcome assessors, clinicians and statisticians were blinded to minimize potential bias.

### 2.9 Outcome measurements

The primary oral function was measured at three time points: baseline, 1 month, and 3 months. The primary outcomes were lip pressure (LP) and tongue pressure (TP). Secondary outcomes included maximum occlusal force (MOF), oral diadochokinesis (OD), repetitive saliva swallowing test (RSST), Simplified Nutritional Appetite Questionnaire (SNAQ), and cough test results. Additionally, the following data were collected: sex, present teeth, functional teeth, maxillectomy, mandibulectomy and glossectomy. The term “present teeth” was defined as erupted teeth, excluding those not in occlusion, stump teeth, or markedly loose teeth. The term of “functional teeth” included present teeth, bridge pontics, artificial teeth on removable dentures, and implant superstructures (12).

#### 2.9.1 Maximum occlusal force

Maximum occlusal force (MOF) was measured with a pressure-sensitive film (Dental Prescale Ⅱ; GC Corp.) (11–13). The participants performed maximum clenching for 3 s in the intercuspal position with the film placed between the maxillary and mandibular dental arch. Then, the film was scanned by a scanning apparatus (GT-X830; Epson Corp.), and the MOF was calculated by the software program (Bite Force Analyzer; GC Corp.).

#### 2.9.2 Lip pressure

Lip pressure (LP) was measured using a LP-measurement device (Lippule-kun; Shofu Inc.) (15, 16). A white button was connected to the device, using 15 mm of dental floss. Then, this button was placed in the anterior region of the oral vestibule. During the measurement, the participants were seated on the dental chair with their heads positioned parallel to the Frankfort horizontal plane. They were given the following instruction: resist traction using the muscle strength of the lips alone, do not resist traction using sucking force (do not make the intraoral pressure negative), and do not bend the head and body forward while resisting.

The assessors stood in front of the participants and measured the lip-seal strength. The participants were instructed to close their lips after confirming that they had placed the button successfully. The LP-measurement device was set at a position on the midline of the face and parallel to the floor to irradiate the subnasale with the indicator light. Then, the button was pulled slowly in the horizontal direction while maintaining the position of the LP-measurement device. The button was pulled until it passed the lips, and the maximum value during this period was indicated on the device. The measurement was performed after confirming that the participant had sufficiently understood the measurement method. Measurements were repeated three times, and the final maximum value was defined as the average value of the three measurements. A rest interval of approximately 30 s was provided between repeated measurements to minimize muscular fatigue.

#### 2.9.3 Tongue pressure

Tongue pressure (TP) was measured by a TP-measurement device (TPM-01; JMS Co. Ltd.) (14). The participants were instructed to place the balloon in their mouth and hold the plastic pipe with their anterior natural teeth or artificial teeth. Then participants raised their tongues to press the balloon to the hard palate for 5 s with maximum pressure. The maximum tongue pressure was the average value of the three measurements. A rest interval of approximately 30 s was provided between repeated measurements to minimize muscular fatigue.

#### 2.9.4 Oral diadochokinesis

Comprehensive motor speed and dexterity measurements for the tongue and lips were measured as OD (11). The participants were instructed to produce the syllables/pa/,/ta/, and/ka/ repeatedly for 5 s each. The number of syllables produced per second was determined using an automatic counter (Kenkokun Handy; Takei Scientific Instruments Co., Ltd.).

#### 2.9.5 Repetitive saliva swallowing test

The RSST was conducted according to published protocols (17). The first and second finger pads were placed gently on the laryngeal prominence. Then, the participant was asked to swallow saliva, and the evaluator confirmed laryngeal elevation as the swallowing reflex. The point where the laryngeal prominence passed the finger pad and moved further downward before returning to the original position was noted; this was considered to be one swallow. The frequency of voluntary swallows in this manner within 30 s was counted. If the participants were unable to follow instructions due to low consciousness or dementia, they received a score of 0 on this test.

#### 2.9.6 Simplified nutritional appetite questionnaire

Appetite was evaluated using the Japanese version of the Simplified Nutritional Appetite Questionnaire (SNAQ), a four-item screening tool ranging from 4 to 20 points, with lower scores indicating poorer appetite and increased risk of weight loss (18).

#### 2.9.7 Cough test

A solution of 1% w/v citric acid–physiological saline was prepared by mixing 0.2 g food additives (citric acid; Fujifilm Wako Pure Chemical Corp.) and 20 mL saline solution (normal saline; Otsuka Pharmaceutical Factory Inc.) (19). The participants orally inhaled the citric acid–physiological saline solution, using a portable mesh nebulizer (NE-U22; Omron Corp.). Those who coughed within 30 s were classified as negative (normal), while those who did not were classified as positive.

### 2.10 Statistical analysis

All statistical analyses were performed using the analysis software (SPSS ver. 22; IBM Corp,). Kolmogorov–Smirnov testing indicated that LP, TP, and MOF were approximately normally distributed (*P* > 0.05). Therefore, parametric analyses were applied. For descriptive purposes, the mean and standard deviation were calculated. For between-group comparisons, Fisher's exact test was used for dichotomous variables, and baseline characteristics were summarized descriptively, and between-group differences were assessed using appropriate statistical tests. The chi-square test was used to test differences in the RSST and the cough test. Spearman's correlation coefficient was employed to assess the relationship between total training time and oral function. Two-tailed *P* values < 0.05 were considered to indicate statistical significance. The primary analysis was planned according to the intention-to-treat principle, including all randomized participants in the analysis according to their assigned groups.

## 3 Results

The recruitment of participants commenced in December 2022 and ended in January 2023. To control for seasonal effects, the trial was scheduled for a period of 3 months. The study flow diagram was prepared in accordance with the CONSORT 2010 guidelines. Based on the inclusion and exclusion criteria, 24 consecutive participants were recruited for the trial (Figure 1). After the introduction of the research program, 4 patients declined to participate. The remaining 20 participants were randomized into the control group (10 participants) and the exercise group (10 participants). Finally, 9 participants in the exercise group (1 dropout: difficult to continue) and 8 participants in the control group (2 dropouts: difficult to visit and continue) finished the analysis, the overall duration of follow-up was 85%. No adverse events or intervention-related complications were reported during the 3-month intervention period. No participants discontinued the intervention because of an adverse event related to the exercise program.

**Figure 1:**
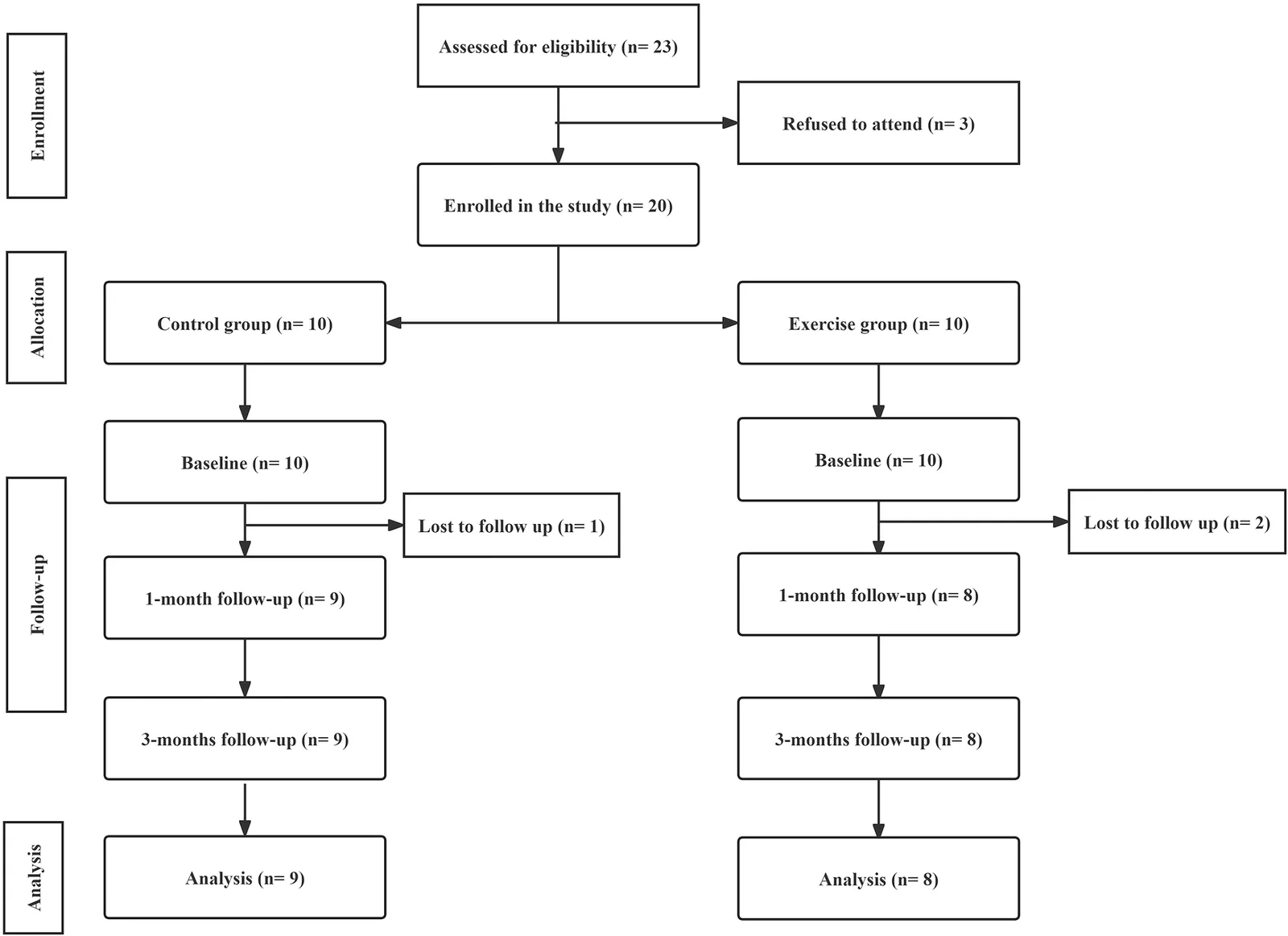
CONSORT 2010 flow diagram of participant progress

Baseline demographic, clinical, treatment, and oral function characteristics are summarized in Table 1, including disease type, surgical characteristics, treatment history, and baseline oral function to facilitate assessment of comparability between the two groups. In addition to demographic variables, Table 1 includes disease type, surgical characteristics, and treatment history to facilitate assessment of baseline comparability between the two groups. The exercise group had significantly more occlusal units without dentures than the control group (*P* = 0.04). No statistically significant differences were observed in the remaining baseline oral function variables. However, a relatively large numerical difference in baseline maximum occlusal force was observed between the groups (430.77 ± 221.74 N vs. 268.52 ± 107.13 N). Although this difference did not reach statistical significance, the magnitude of the numerical difference should be considered when interpreting the treatment effect. Given the small sample size and substantial inter-individual variability, baseline imbalance may have influenced the observed changes in maximum occlusal force. Oral function outcomes, including within-group changes over time and between-group comparisons of change scores, are summarized in Table 2 and the trajectories of maximum occlusal force and lip pressure over the study period are illustrated in Figures 2, 3.

**Table 1:** Baseline demographic, clinical, treatment, and oral function characteristics of the study participants.

Baseline	Total	Control group	Exercise group	*P* value
	mean (SD)	mean (SD)	mean (SD)	
Female (%)	47	25	67	0.10
Age (years)	71.16 (6.02)	73.00 (4.31)	70.67 (7.28)	0.44
Present teeth	15.76 (8.27)	11.75 (9.18)	19.33 (5.70)	0.07
Functional teeth	26.06 (6.81)	27.63 (1.19)	24.67 (9.31)	0.38
Occlusal units without dentures	4.88 (4.09)	2.75 (2.92)	6.78 (4.18)	0.04^a^
Occlusal units with dentures	13.71 (0.69)	13.63 (0.74)	13.78 (0.67)	0.66
MOF (N)	332.01 (179.08)	430.77 (221.74)	268.52 (107.13)	0.12
LP (N)	8.77 (3.04)	10.47 (3.65)	7.72 (2.06)	0.14
TP (kPa)	26.34 (9.78)	30.17 (10.07)	24.35 (9.64)	0.39
OD of/pa/ (times/s)	5.29 (1.45)	5.26 (2.03)	5.44 (0.76)	0.67
OD of/ta/ (times/s)	5.51 (0.98)	5.94 (0.81)	5.16 (1.03)	0.12
OD of/ka/ (times/s)	4.93 (1.03)	4.91 (1.17)	4.87 (0.96)	0.80
SNAQ	14.88 (1.50)	14.86 (2.05)	15.00 (0.87)	0.74
RSST—negative (%)	82.35	87.50	77.78	0.63
Cough test—negative (%)	88.24	75.00	100.00	0.17

SD, standard deviation; MOF, maximum occlusal force; LP, lip pressure; OD, oral diadochokinesis; TP, tongue pressure; SNAQ, Simplified Nutritional Appetite Questionnaire; RSST, Repetitive Saliva Swallowing Test.

a Significant difference was detected.

**Table 2:** Oral function outcomes at baseline, 1 month, and 3 months, including within-group comparisons and between-group differences in change scores.

Variable	Group	Baseline mean (SD)	1 month mean (SD)	3 months mean (SD)	P (Baseline vs. 1 month)	P (Baseline vs. 3 months)	*Δ* Baseline–3 months mean (SD)	Between-group P
MOF (N)	Control	430.77 (221.74)	390.24 (254.70)	340.43 (340.01)	0.58	0.37	−63.00 (186.48)	0.02*
	Exercise	268.52 (107.13)	329.01 (173.72)	417.42 (170.64)	0.19	0.01*	148.91 (138.79)	
LP (N)	Control	10.47 (3.65)	11.04 (3.05)	11.18 (4.28)	0.37	0.11	1.23 (1.91)	0.77
	Exercise	7.72 (2.06)	8.95 (2.03)	9.17 (1.91)	0.02*	0.004*	1.45 (1.09)	
TP (kPa)	Control	30.17 (10.07)	28.74 (9.42)	27.44 (9.24)	0.43	0.69	−1.15 (7.79)	0.25
	Exercise	24.35 (9.64)	24.08 (8.97)	26.89 (9.09)	0.79	0.13	2.53 (4.56)	
OD /pa/	Control	5.26 (2.03)	4.97 (1.50)	5.25 (1.84)	0.45	0.75	0.13 (1.06)	0.34
	Exercise	5.44 (0.76)	5.62 (0.94)	5.93 (0.75)	0.21	0.002*	0.49 (0.32)	
OD /ta/	Control	5.94 (0.81)	5.40 (1.34)	5.70 (1.18)	0.10	0.48	−0.20 (0.76)	0.09
	Exercise	5.16 (1.03)	5.33 (1.08)	5.80 (1.29)	0.24	0.12	0.64 (1.11)	
OD /ka/	Control	4.91 (1.17)	5.00 (0.96)	4.90 (1.66)	0.83	0.82	−0.10 (1.18)	0.35
	Exercise	4.87 (0.96)	5.00 (1.06)	5.24 (1.17)	0.44	0.21	0.38 (0.84)	
SNAQ	Control	14.86 (2.05)	15.00 (2.16)	14.63 (2.26)	0.36	0.84	−0.13 (1.73)	0.40
	Exercise	15.00 (0.87)	16.00 (1.23)	15.44 (0.88)	0.08	0.17	0.44 (0.88)	

*Significant difference was detected.

**Figure 2:**
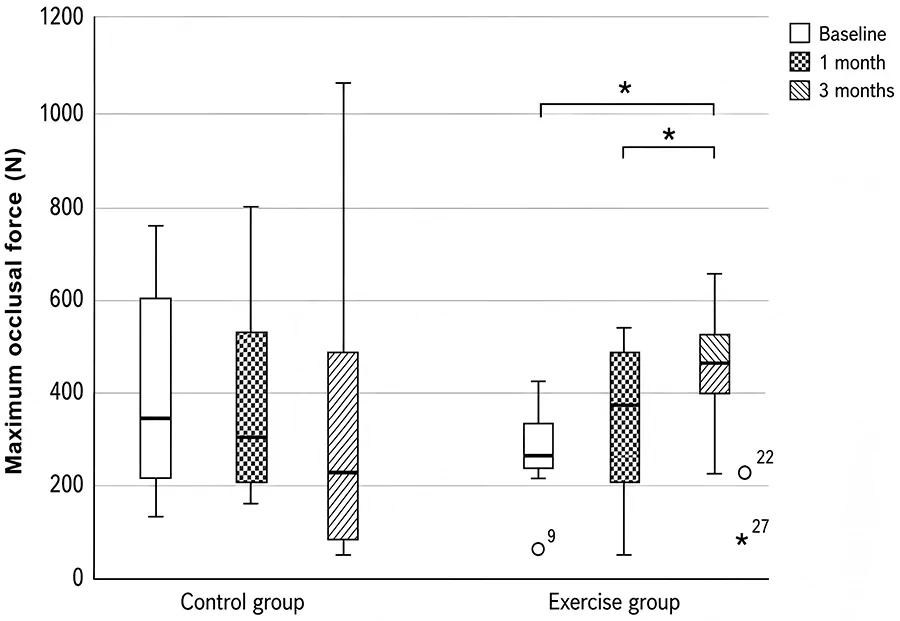
Changes in maximum occlusal force during the study period.

**Figure 3:**
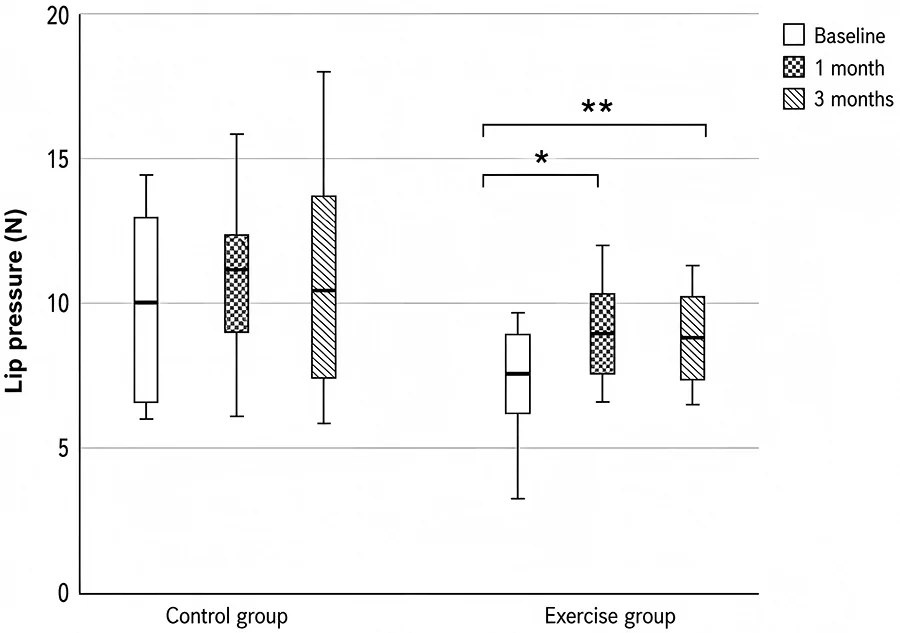
Changes in lip pressure during the study period.

In the exercise group, lip pressure increased from 7.72 ± 2.06 N at baseline to 8.95 ± 2.03 N after 1 month (*P* = 0.02) and to 9.17 ± 1.91 N after 3 months (*P* = 0.004) (Table 2; Figure 3). Maximum occlusal force increased from 268.52 ± 107.13 N at baseline to 417.42 ± 170.64 N after 3 months (*P* = 0.01), with an additional increase between 1 month and 3 months (329.01 ± 173.72 N to 417.42 ± 170.64 N; *P* = 0.01) (Table 2; Figure 2). Oral diadochokinesis for/pa/ increased from 5.44 ± 0.76 to 5.93 ± 0.75 times/s after 3 months (*P* = 0.002) (Table 3). No significant within-group changes were observed in the control group. Between-group comparisons of change scores demonstrated significant differences for oral diadochokinesis/ta/ between baseline and 1 month (*P* = 0.03) and for maximum occlusal force between baseline and 3 months (*P* = 0.02) (Table 2).

**Table 3:** Correlation between total training time and oral function.

Spearman's rho		MOF (N)	LP (N)	TP (kPa)	OD of /pa/ (times/s)	OD of /ta/ (times/s)	OD of /ka/ (times/s)	SNAQ
Total training time (min)	Correlation Coefficient	.481^a^	.713^b^	.274	.643^b^	.557^b^	.492^b^	.337
	*P* value (2-tailed)	.011	<.001	.167	<.001	.003	.009	.085

MOF, maximum occlusal force; LP, lip pressure; OD, oral diadochokinesis; TP, tongue pressure; SNAQ, Simplified Nutritional Appetite Questionnaire.

a Correlation is significant at the 0.05 level (2-tailed).

b Correlation is significant at the 0.01 level (2-tailed).

Correlations between total training time and changes in oral function are presented in Table 3. Greater total training time was associated with larger improvements in maximum occlusal force (*ρ* = 0.481, *P* = 0.011), lip pressure (*ρ* = 0.713, *P* < 0.001), oral diadochokinesis/pa/ (*ρ* = 0.643, *P* < 0.001),/ta/ (*ρ* = 0.557, *P* = 0.003), and/ka/ (*ρ* = 0.492, *P* = 0.009) (Table 3).

## 4 Discussion

The present pilot randomized controlled trial investigated the feasibility and potential effects of a music-assisted oral exercise program on oral function in patients with maxillofacial defects. The principal findings were that participants in the exercise group demonstrated significant improvements in lip pressure, maximum occlusal force, and oral diadochokinesis after 3 months, whereas no significant improvements were observed in the control group. Furthermore, greater training time was associated with larger functional gains. These findings suggest that the combined music-and-exercise program is feasible and may improve selected measures of oral function in this patient population. However, because of the small sample size and exploratory nature of the study, these findings should be interpreted cautiously until confirmed in larger randomized trials.

The role of music warrants particular consideration because it represents the novel component of the intervention. Previous studies have demonstrated that music can facilitate motor learning through rhythmic entrainment and improve adherence by increasing enjoyment and intrinsic motivation. Satoh et al. reported beneficial effects of music-based exercise on cognitive and psychological outcomes (9), while Feiss et al. observed enhanced exercise engagement when music accompanied physical activity (10). The present findings are consistent with previous reports that music may facilitate participation in exercise programs (9, 10). However, because this study did not include an exercise-only comparison group, the effects of music cannot be distinguished from those of exercise. Therefore, the observed improvements should be attributed to the combined intervention rather than to music itself.

The exercise group showed progressive improvements in oral function over the 3-month intervention period, particularly for maximum occlusal force and lip pressure (Figures 2, 3), whereas the control group showed relative stability or slight decline. Although a significant portion of the exercise regimen focused on pronunciation, the regular practice of mouth-opening also played a crucial role in simultaneously training various oral muscles. This aspect may explain the observed enhancement in MOF. Minakuchi et al. previously reported that low occlusal force can reduce the intake of essential nutrients from vegetables, fruits, antioxidant vitamins, and dietary fiber (11). Consequently, the improvement in MOF observed in the present study likely contributed to enhanced food intake abilities, ultimately higher SNAQ scores. The exercise, which included consonant pronunciation drills such as repeating/pa/,/ta/, and/ka/, were designed to target specific aspects of oral function, including lip movement, the anterior and posterior regions of the tongue, and enhance the tongue-lip motor function, this was also supported by the study of Hori et al. (2). In the TP index of oral hypofunction defined by the Japanese Society of Gerodontology, the cutoff value of TP is 30 kPa (11). Although tongue pressure values remained below the established threshold after the intervention, a modest increase was observed at 3 months. The clinical significance of this change remains uncertain and should be evaluated in larger studies. Notably, at baseline, 2 participants were identified as having a high risk of dysphagia. After 3 months of exercise, RSST values for all patients became negative, confirming the positive impact of the exercise on the swallowing function. In contrast, the RSST results of the patients in the control group declined over time, emphasizing the need for proactive attention from prosthodontists. The cough test has been found to be a simple and noninvasive method for assessing maxillofacial defect patients when compared with more invasive techniques such as videofluoroscopic and videoendoscopic examination. The results of the cough test provided reassurance by indicating that none of the patients experienced silent aspiration, thereby emphasizing the significance of age as a risk factor, particularly for older adults with chronic diseases (19). Therefore, it is essential to continue monitoring patients in the future. In summary, the improvements in oral function observed in this study can be attributed not only to pronunciation exercises but also to the inclusion of mouth-opening movements that effectively trained various oral muscles. These improvements may support oral functional performance; however, nutritional outcomes, quality of life, and other patient-reported outcomes were not evaluated in this study.

The positive correlations between total training time and changes in maximum occlusal force, lip pressure, and oral diadochokinesis suggest that greater participation in the intervention was associated with larger functional improvements. However, causality cannot be inferred because of the exploratory design and limited sample size.

Several limitations of this study should be acknowledged. First, the study included patients with both HNC and CLP, who differ in etiology, surgical treatment, adjuvant therapy, and oral functional impairment. Because randomization was stratified only by age and sex, rather than by defect type or treatment history, baseline imbalance between groups occurred, particularly for occlusal units without dentures. In addition, numerical differences in baseline maximum occlusal force were observed. Although the baseline difference in maximum occlusal force was not statistically significant, its magnitude may have influenced treatment comparisons in this small pilot study. Therefore, baseline maximum occlusal force should be considered when interpreting the between-group treatment effect. Therefore, the findings should be interpreted cautiously, and future studies should employ stratified randomization based on defect type and major treatment characteristics. Second, participants in the exercise group were allowed to select different exercise durations (6, 9, or 24 min) and choose their preferred songs. While this approach was intended to improve motivation and adherence, it introduced variability in exercise exposure and may have contributed to differences in treatment response. Third, the intervention combined multiple components, including vocalization exercises, consonant articulation training, singing, and limb movements synchronized with music. Consequently, the specific contribution of music to the observed improvements could not be isolated. Future studies should include a comparison group receiving identical exercises without music to clarify the unique effects of the musical component. Although the calculated sample size was 24 participants, the original calculation did not include an explicit allowance for attrition, and only 17 participants completed the study. The resulting reduction in sample size may have limited statistical power. The reduced sample size may have limited statistical power and increased the risk of type II error. Therefore, the findings should be interpreted as preliminary. Larger multicenter pilot randomized controlled trials with adequate statistical power, intention-to-treat analyses, and longer follow-up periods are needed to confirm the effectiveness of music-assisted oral rehabilitation in patients with maxillofacial defects. Detailed information regarding surgical extent and adjuvant treatment was unavailable for baseline comparison, which limits the assessment of baseline comparability and the interpretation of treatment effects. Because these clinical characteristics may influence oral function, the absence of these data limits the assessment of baseline comparability and may have introduced residual confounding. Future studies should prospectively collect these variables and incorporate them into the randomization strategy and statistical analyses. Accordingly, the findings should be considered preliminary. Larger multicenter pilot randomized controlled trials with adequate statistical power, intention-to-treat analyses, an exercise-only comparison group, and longer follow-up periods are needed to confirm these findings and clarify the specific contribution of music to the intervention.

## 5 Conclusions

This exploratory pilot randomized trial suggests that participation in the combined music-and-exercise program may improve maximum occlusal force, lip pressure, and oral diadochokinesis (/pa/) in patients with maxillofacial defects. However, because of the small sample size and the absence of a non-music exercise control group, these findings should be considered preliminary. Larger, adequately powered randomized controlled trials are needed to confirm these findings and clarify the specific contribution of music to the intervention.

## Data Availability

The original contributions presented in the study are included in the article/Supplementary Material, further inquiries can be directed to the corresponding author.
